# Application of War Surgery to Civil Hospital Conditions

**Published:** 1919-01

**Authors:** 


***The Application of the Teachings of War Surgery to Civil Hospital Conditions***. By JOHN A. HARTWELL and ETHAN F. BUTLER. *Surgery, Gynecology and Obstetrics*, October 1918.

The authors consider that many improvements should be made in the conditions of civil hospitals, according to the experience gained in military medical practice.

They consider that, at the beginning of the war, most of the difficulties that arose in the treatment of war wounds would have been easily subdued had the surgeons been more familiar with the process of infection, its development and healing. But, unfortunately, it was formerly supposed that cases of infection in civil practice were obliged to terminate in some degree of permanent disability, and that no form of treatment was available in order to improve the results.

New experience has now been obtained and the needed improvement is along the line of a definite reorganization of the entire hospital plan.

The military situation makes it possible for the surgeon to call to his aid physicists, chemists, pathologists, and bacteriologists. They work effectively and efficiently together, and the same intimate and close co-operation must exist henceforth in the civil hospitals. Both the staffs of surgeons and of laboratory men will profit by close contact.

These staffs must be composed largely of " full time " men. Whatever the financial difficulties of such an organization, this is considered by the authors an absolute necessity. The members of the staff must give their whole time and their undivided attention to the patients within the hospital.

Another improvement, more easily obtainable, would be the equipment within every hospital of a workshop, in which a competent workman would always be at hand who could promptly furnish the apparatus, and suggest ways of meeting all mechanical problems.

The authors express the hope that a certain number of principles underlying the treatment of war injuries may be applied in civil practice, and consider that : first, severe traumata, badly contaminated with bacteria, need not go on to active infection and suppuration; second, these sequelae are largely the result of certain physical as well as biological factors; third, if these sequelae do supervene, they may be largely controlled by removal of physical factors, rather than by killing the bacteria; fourth, aseptic surgery is not confined to clean operative wounds made through carefully prepared tissues.

The authors have made quite a special study of the chlorine antiseptics, and the Carrel-Dakin method. Thanks to the application of this method the worst suppuration can practically be controlled in the hospital ward. The greatest value of the hypochlorite solution does not seem to lie in its bactericidal power. Its great efficiency is due to another more important attribute.

Sodium hypochlorite in a watery solution of 0.45 per cent. to 0.5 per cent. is strongly proteolytic. It is more potent in dissolving necrotic tissues than living protoplasm, although it can attack the latter. The reaction is by no means specific. The protoplasm of bacteria is attacked with no more avidity than the protein on which the bacteria live. But the living human tissue has a strong defense against digestion. When the hypochlorite comes in contact with it, an immediate response is the outpouring of tissue fluids containing an abundance ofprotein in solution. This takes up instantly the available chlorine and the concentration falls below the active limit.

If the wound under treatment contains bacteria, more or less destroyed leukocytes and inflammatory debris, the first reaction is with these substances. Thus the surface of the wound becomes clean.

This explains why it is that only when the acute suppurative stage of the wound has been controlled, the surrounding skin need be protected; and also why skin burns developing during the Carrel-Dakin treatment usually remain superficial. The superficial burn immediatly pours out albuminous fluids, hence the solution immediatly loses its power to burn.

That is also the reason why recently injured joints, in which suppuration has not taken place, should not be exposed to hypochlorite; the response in tissue fluids may not be sufficient to protect the joint from erosion. But a joint already infected, and filled with purulent exudate, must be given the hypochlorite treatment.

These considerations have induced the authors to apply the Carrel-Dakin treatment to empyema. The later practice has been the usual rib resection and evacuation of the pus, and the insertion of tubes through which Dakin solution is slowly run into the cavity under very low pressure. Hourly or two-hourly irrigations are instituted. In some cases, thanks to this treatment, well developed empyemata have been rendered aseptic in five to nine days with secondary suture of the chest resulting in complete healing.

The properties of Dakin solution may be summed up as follows :

(1) It is a powerful digestant of protein substances.

(2) This includes the protein of bacteria, hence it is a bactericide.

(3) It stimulates wound surfaces to pour out albuminous materials and leukocytes; hence it still further destroys bacteria and draws out locally the body defenses.

Dichloramine T. does not prove so efficient as hypochlorite solution. It lacks an appreciable proteolytic power and simply acts on the wound surface because of its bactericidal properties.

